# Supplementary material for: Enhanced performance of in-plane transition metal dichalcogenides monolayers by configuring local atomic structures
Source: Nat Commun. 2020 May 7;11:2253. doi: 10.1038/s41467-020-16111-0 (PMC7205865; doi:10.1038/s41467-020-16111-0)
Supplement: Supplementary file 1 — Supplementary Information [file 41467_2020_16111_MOESM1_ESM.pdf]

Supplementary Information

Enhanced Performance of In-Plane Transition Metal Dichalcogenides Monolayers by Configuring Local Atomic Structures

Zhou et al.

Supplementary Figures

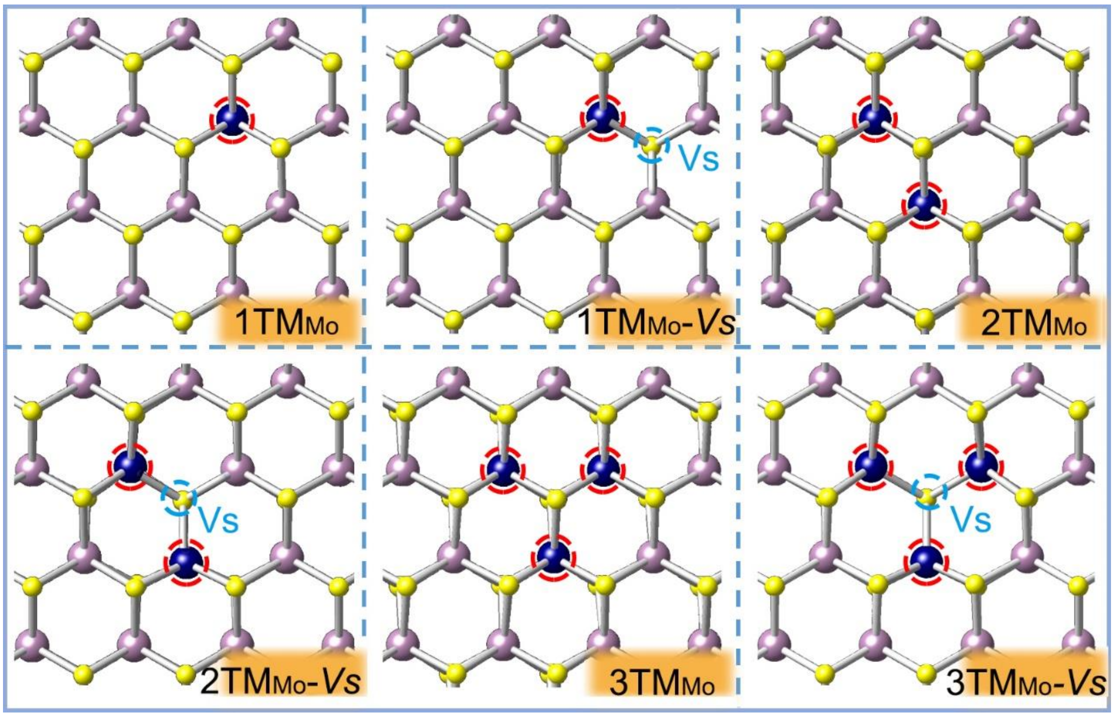

Supplementary Figure 1. Possible local configurations from sole TM atom to TM clusters with sulfur vacancy when TM atoms are Co, Fe, V, and Cr in period-IV, where TM is transition metal highlighted in red circle and sulfur vacancy in blue circle.

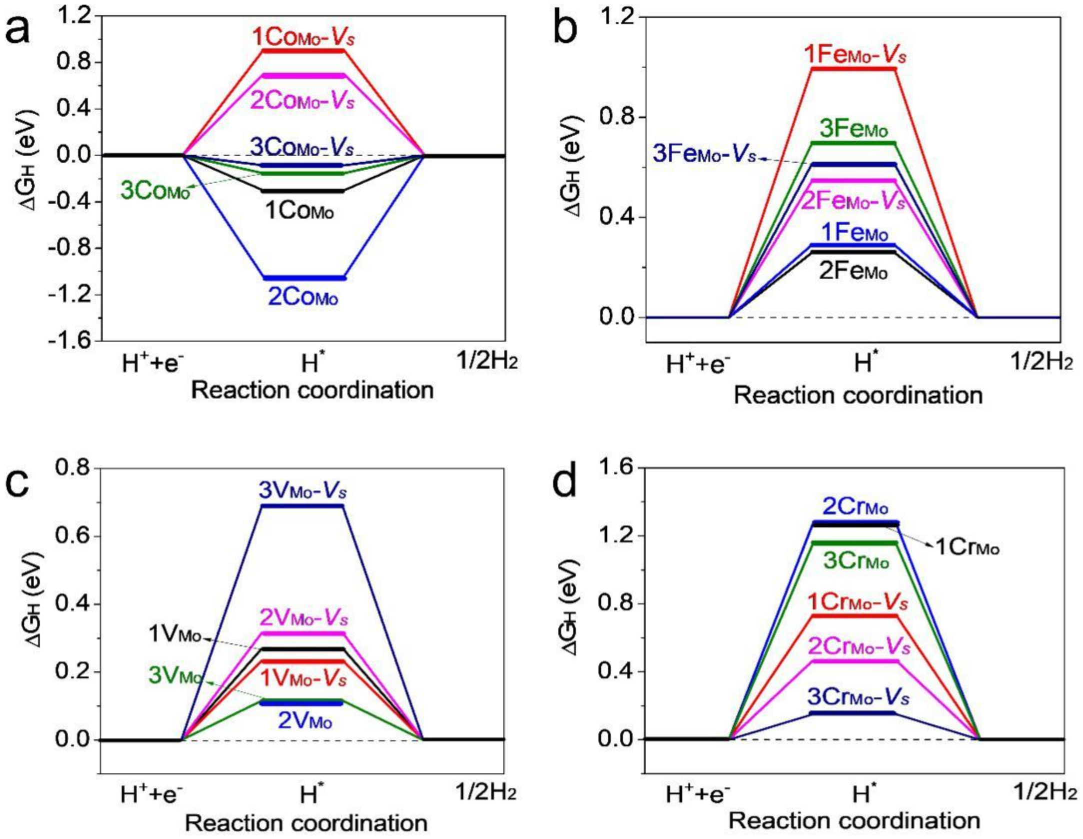

Supplementary Figure 2. The calculated hydrogen adsorption free energies (ΔG_H). ΔG_H based on the proposed local configurations mentioned of (a) Co-, (b) Fe, (c) V-, and (d) Cr-containing MoS_2.

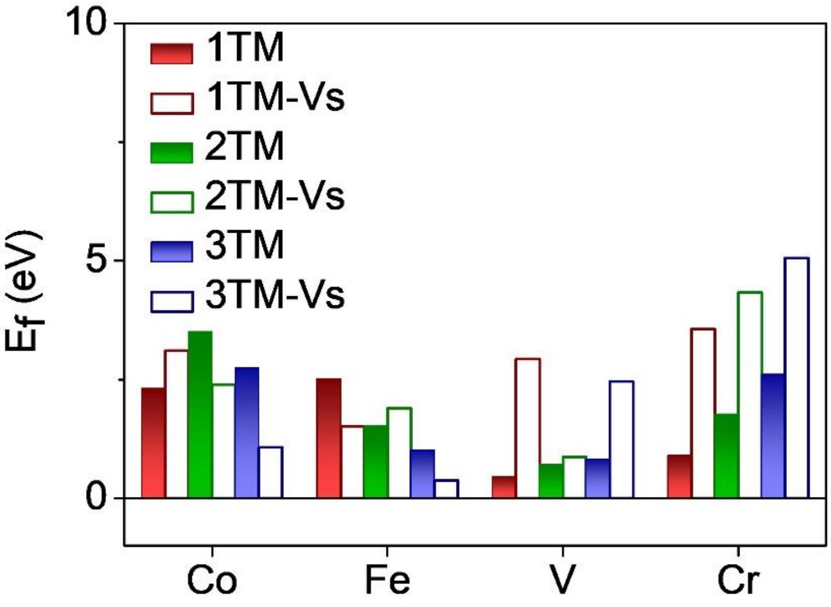

Supplementary Figure 3. The formation energies of proposed local configurations. The formation energies are calculated as $E_f = E(xTM\_nVs) + xE(Mo) + nE(S) - E(MoS_2) - xE(TM)$.

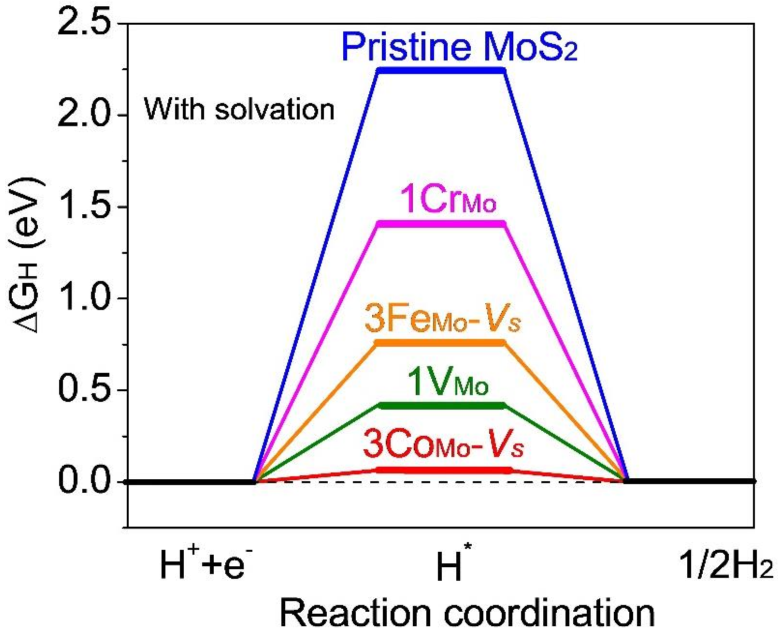

Supplementary Figure 4. The calculated free energy diagram of various configurations and pristine MoS_2 with solvation effect.

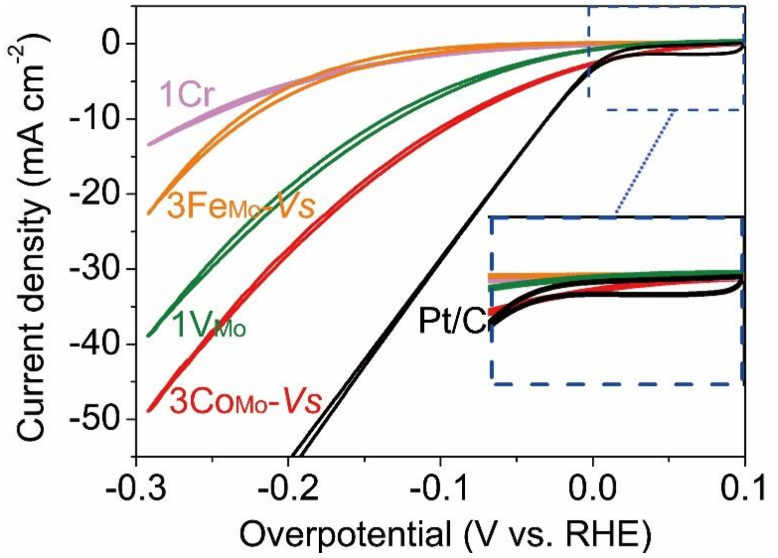

Supplementary Figure 5. The first CVs of the 3Co$_{Mo}$-*Vs*, 1V$_{Mo}$, 3Fe$_{Mo}$-*Vs*, 1Cr$_{Mo}$ and Pt/C for the HER. Compared to Pt/C, there is nearly no hysteresis loop in the CVs of the configured MoS_2 samples in the non-Faradaic range (dashed box). It implies minimized double layer contribution for the configured samples.

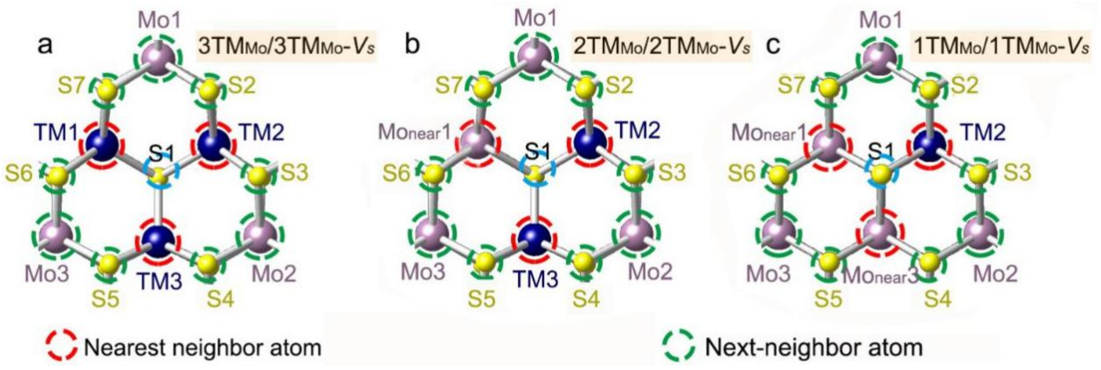

Supplementary Figure 6. The illustration of the nearest neighbor atoms and the next-neighbor atoms. The nearest neighbor atoms and the next-neighbor atoms in (a) $3TM_{Mo}/3TM_{Mo}$-*Vs* and (b) $2TM_{Mo}/2TM_{Mo}$-*Vs*, and (c) $1TM_{Mo}/1TM_{Mo}$-*Vs* system.

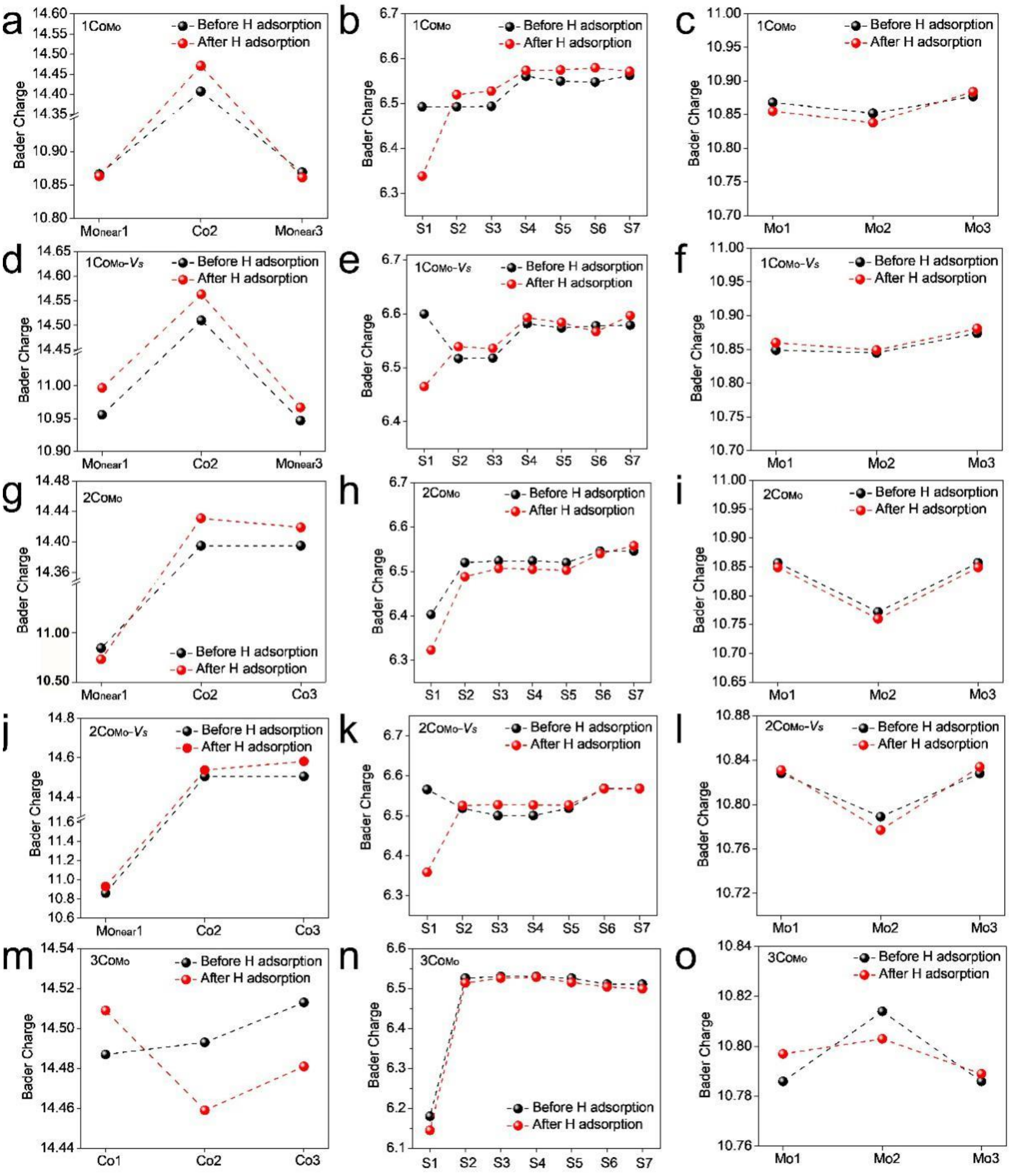

Supplementary Figure 7. Bader charge analysis of nCo (n= 1, 2, 3) doped MoS_2 with or without S vacancy structures before and after H adsorption. The Bader charge changes of the nearest Co atom/Mo atoms (a, d, g, j, m) around the adsorption site S1 atom, the S1 and the next-neighbor S atoms (b, e, h, k, n) and the next-neighbor Mo atoms (c, f, i, l, o).

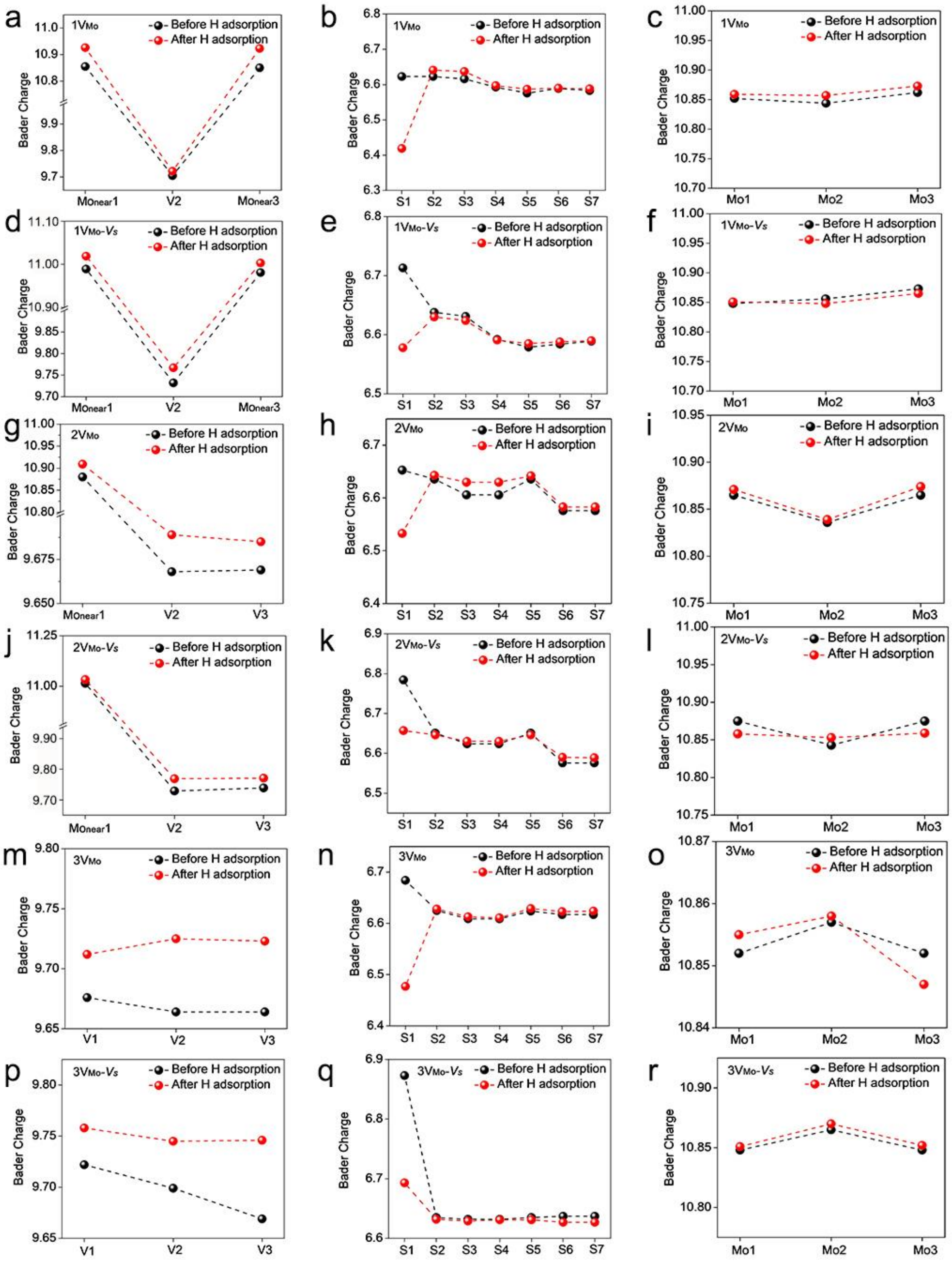

Supplementary Figure 8. Bader charge analysis of nV (n= 1, 2, 3) doped MoS_2 with or without S vacancy structures before and after H adsorption. The Bader charge changes of the nearest V atom/Mo atoms (a, d, g, j, m, p) around the adsorption site S1 atom, the S1 and the next-neighbor S atoms (b, e, h, k, n, q) and the next-neighbor Mo atoms (c, f, i, l, o, r).

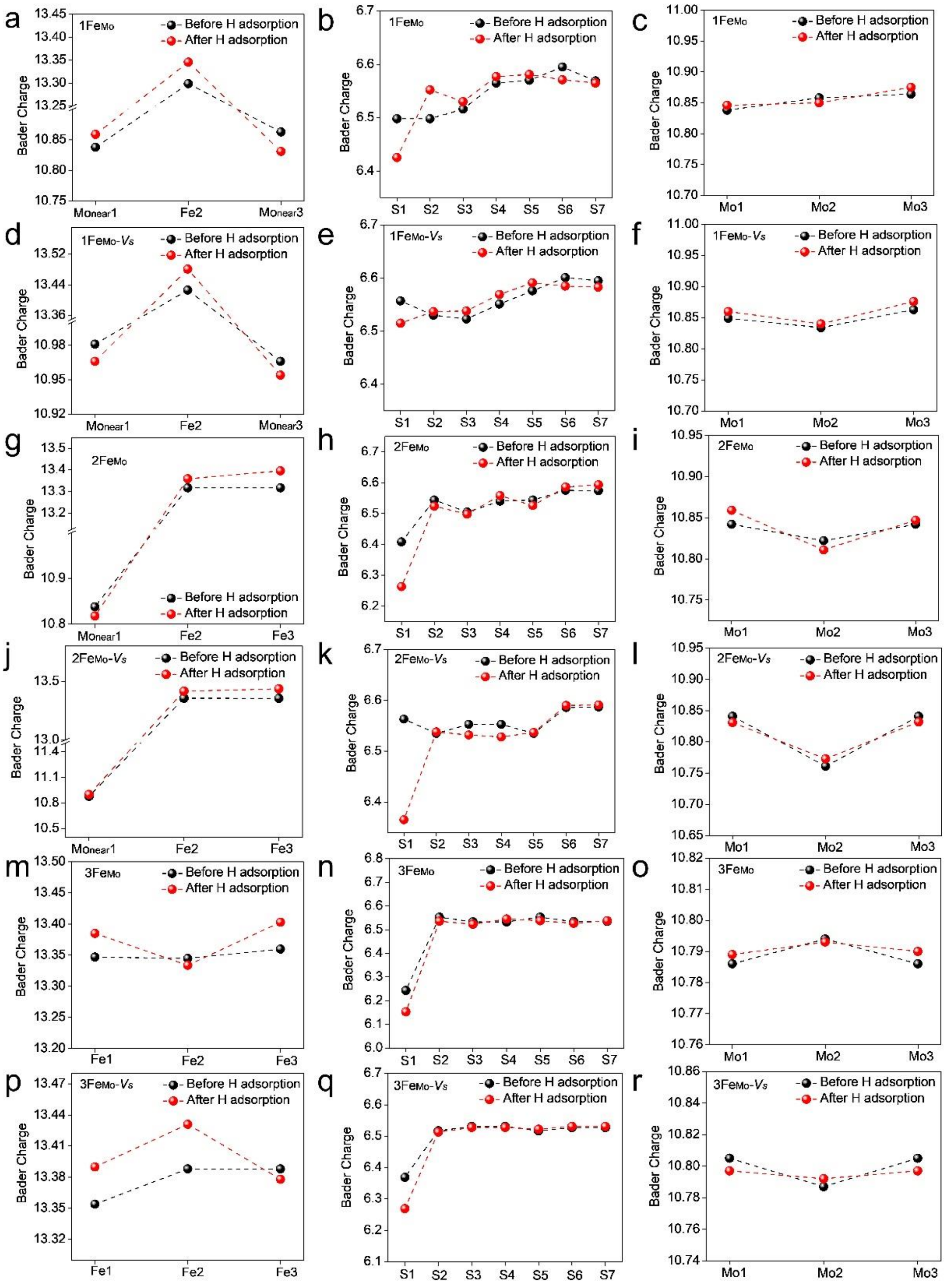

Supplementary Figure 9. Bader charge analysis of nFe (n= 1, 2, 3) doped MoS_2 with or without S vacancy structures before and after H adsorption. The Bader charge changes of the nearest Fe atom/Mo atoms (a, d, g, j, m, p) around the adsorption site S1 atom, the S1 and the next-neighbor S atoms (b, e, h, k, n, q) and the next-neighbor Mo atoms (c, f, i, l, o, r).

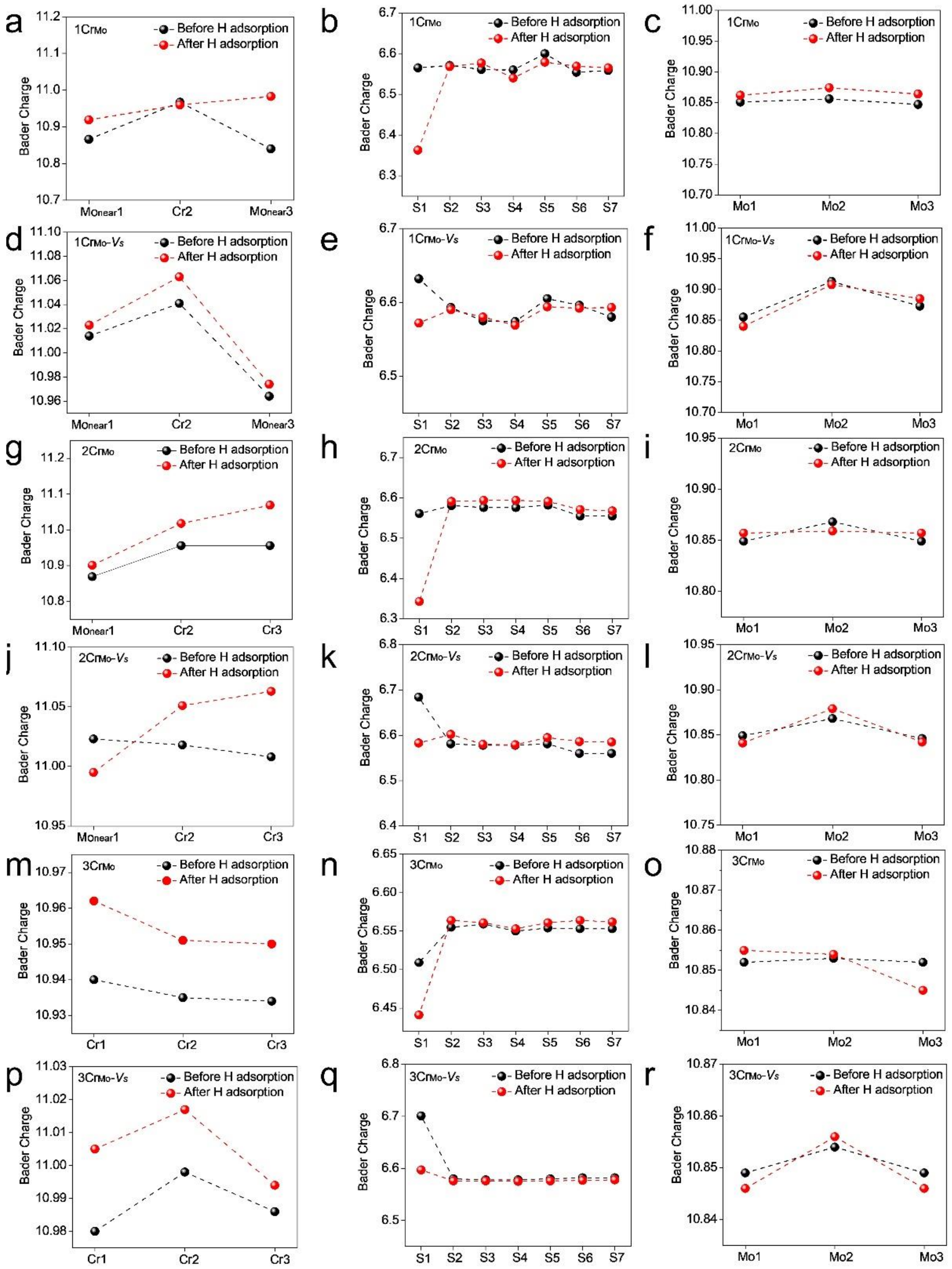

Supplementary Figure 10. Bader charge analysis of nCr (n= 1, 2, 3) doped MoS_2 with or without S vacancy structures before and after H adsorption. The Bader charge changes of the nearest Cr atom/Mo atoms (a, d, g, j, m, p) around the adsorption site S1 atom, the S1 and the next-neighbor S atoms (b, e, h, k, n, q) and the next-neighbor Mo atoms (c, f, i, l, o, r).

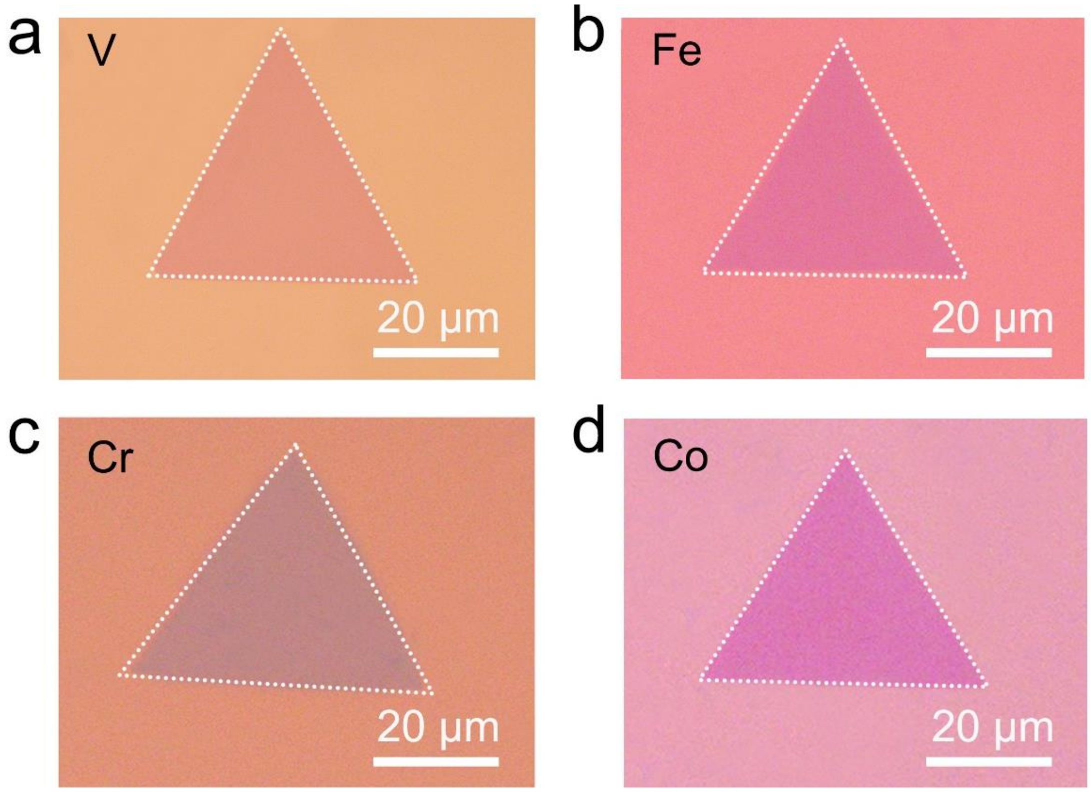

Supplementary Figure 11. Optical images of configured MoS_2. Optical images of (a) V-, (b) Fe-, (c) Cr-, and (d) Co-containing monolayer MoS_2.

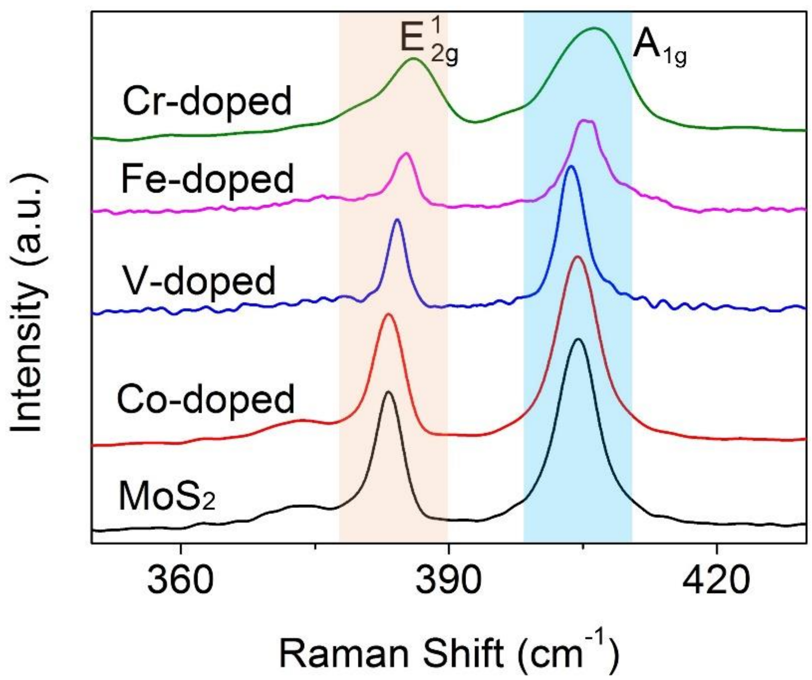

Supplementary Figure 12. Raman spectrum of V-, Fe-, Cr-, Co-containing MoS_2 monolayer and pristine MoS_2 monolayer.

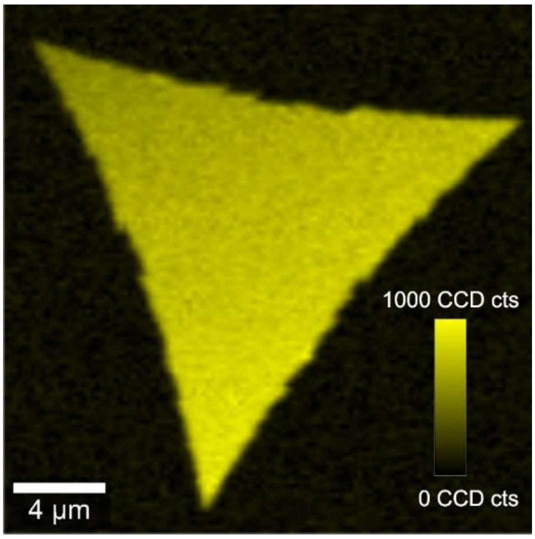

Supplementary Figure 13. Raman mapping of Co-containing MoS_2 monolayer with Co doping concentration of 3.8 %.

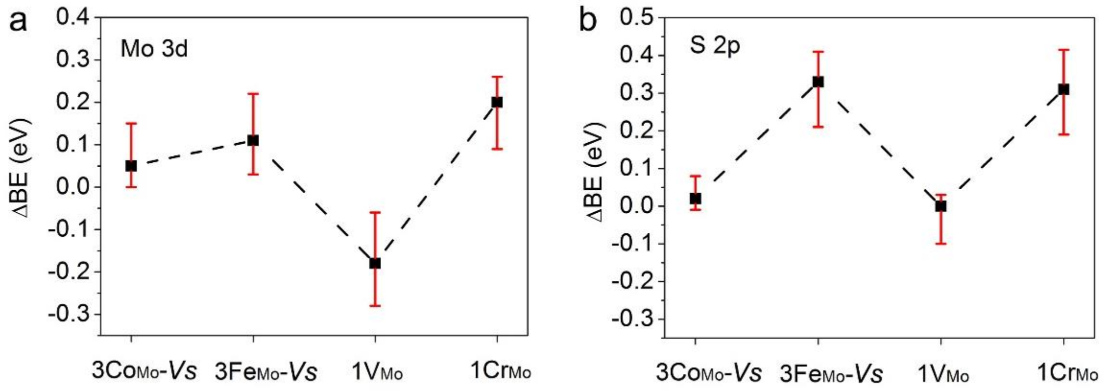

Supplementary Figure 14. The errors of ΔBE for (a) Mo 3d and (b) S 2p of the $3Co_{Mo}$-*Vs*, $3Fe_{Mo}$-*Vs*, $1V_{Mo}$, and $1Cr_{Mo}$ samples.

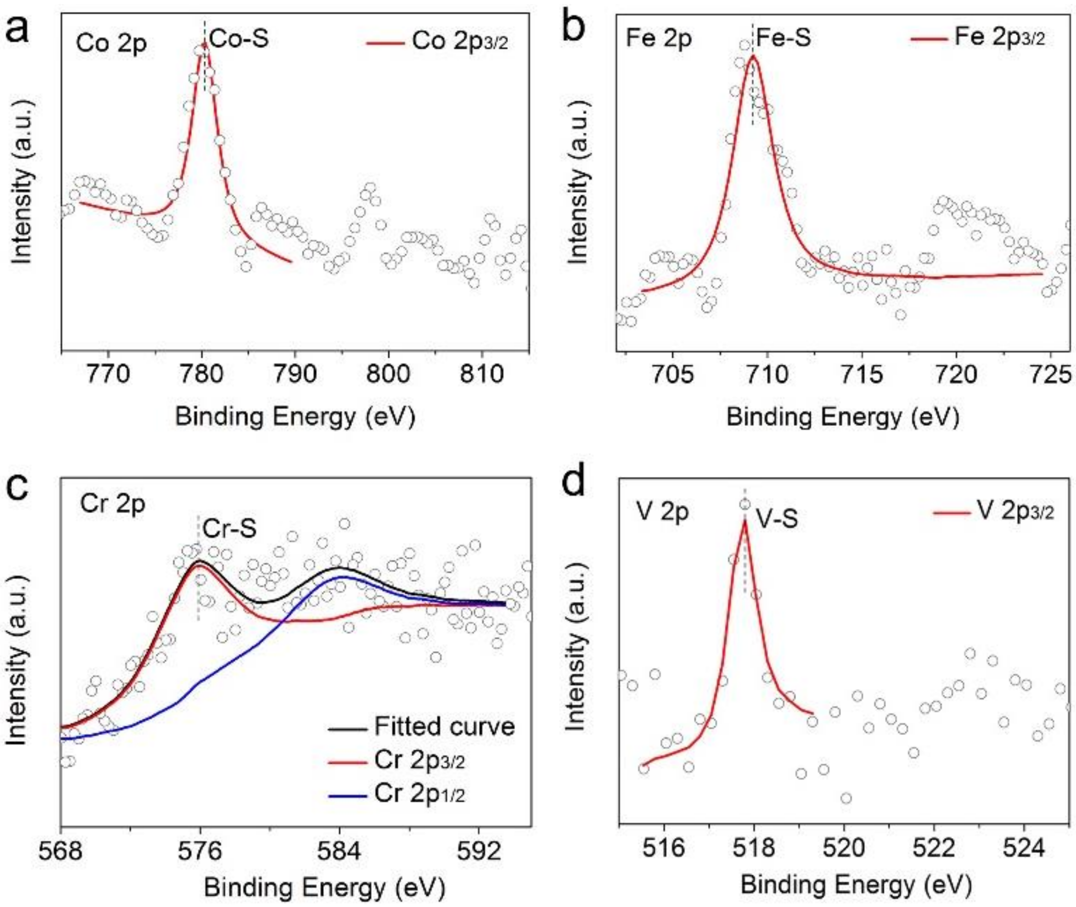

Supplementary Figure 15. The metal 2p of XPS spectra. The corresponding high-resolution metal 2p spectra in the (a) $3Co_{Mo}$-*Vs*, (b) $3Fe_{Mo}$-*Vs*, (c) $1Cr_{Mo}$, and (d) $1V_{Mo}$.

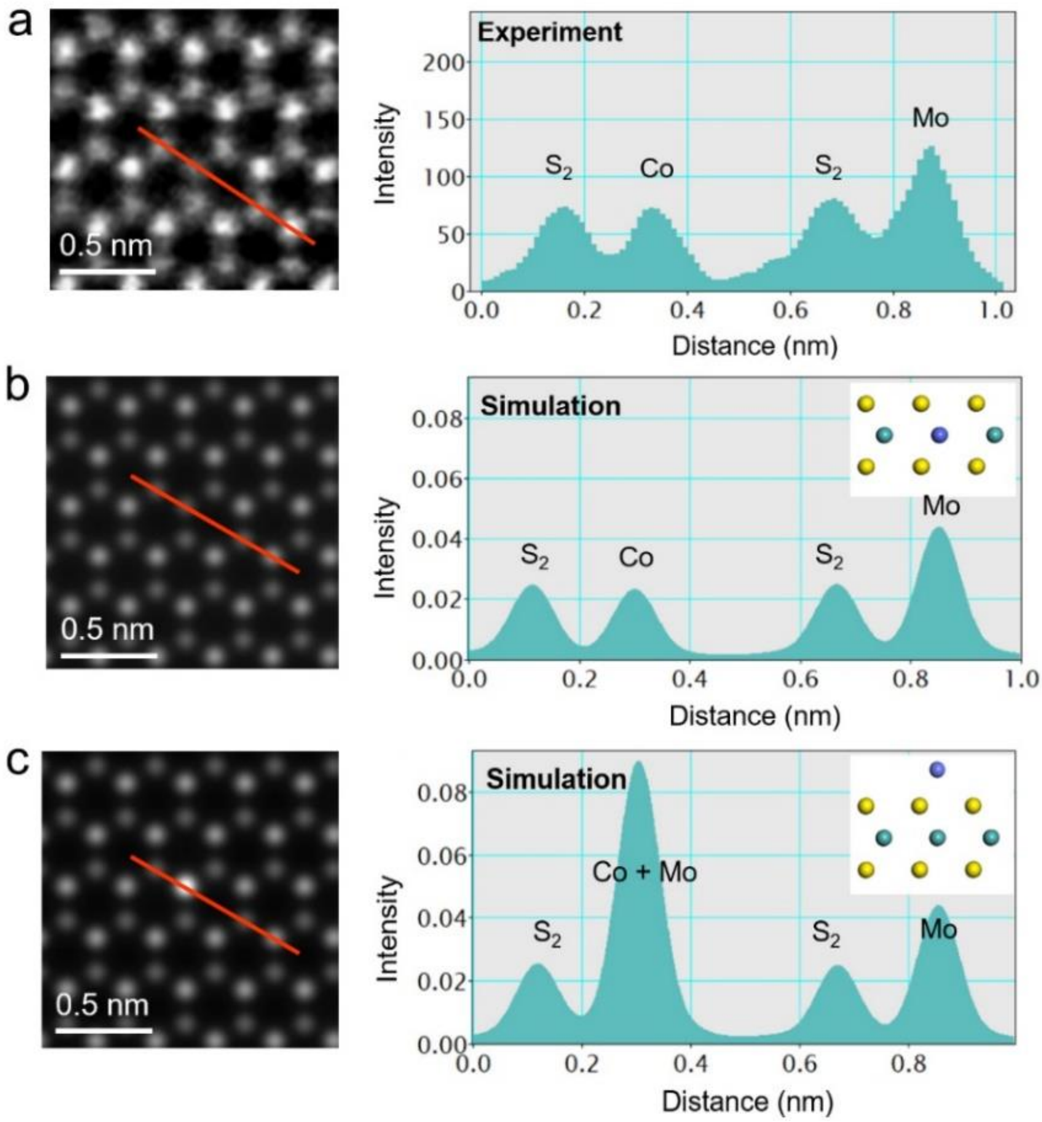

Supplementary Figure 16. Line intensity profile of the single isolated Co dopant. (a) Experimental STEM image of the single isolated Co dopant with its line intensity profile along the highlighted red line. (b, c) Simulated STEM image of the substitutional Co dopant (b) and Co adatom on top of the Mo site (c). From the intensity profile, the simulation of the substitutional dopant configuration agrees well with the experiment, thus excluding the adatom configuration.

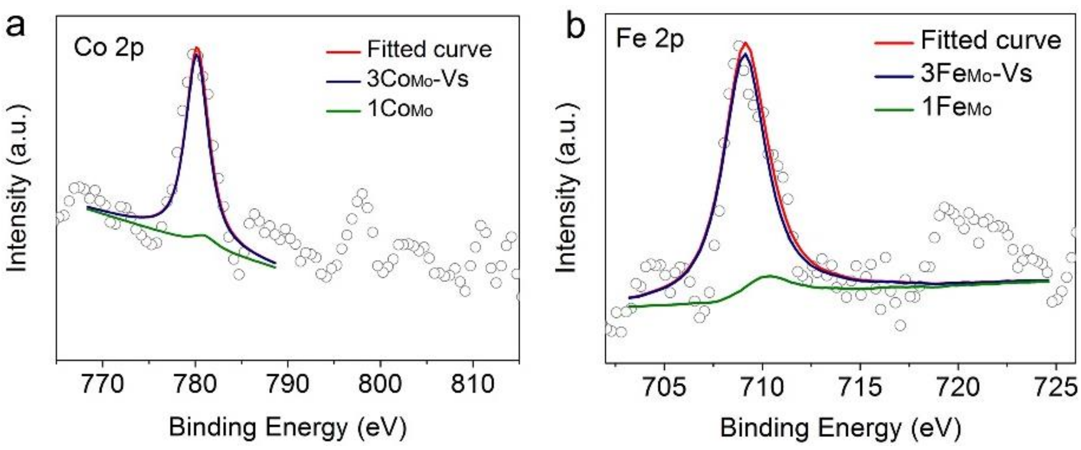

Supplementary Figure 17. The Co and Fe 2p of XPS spectra. The fitted curves of (a) Co 2p of the $3Co_{Mo}$-*Vs* and (b) Fe 2p of the $3Fe_{Mo}$-*Vs*.

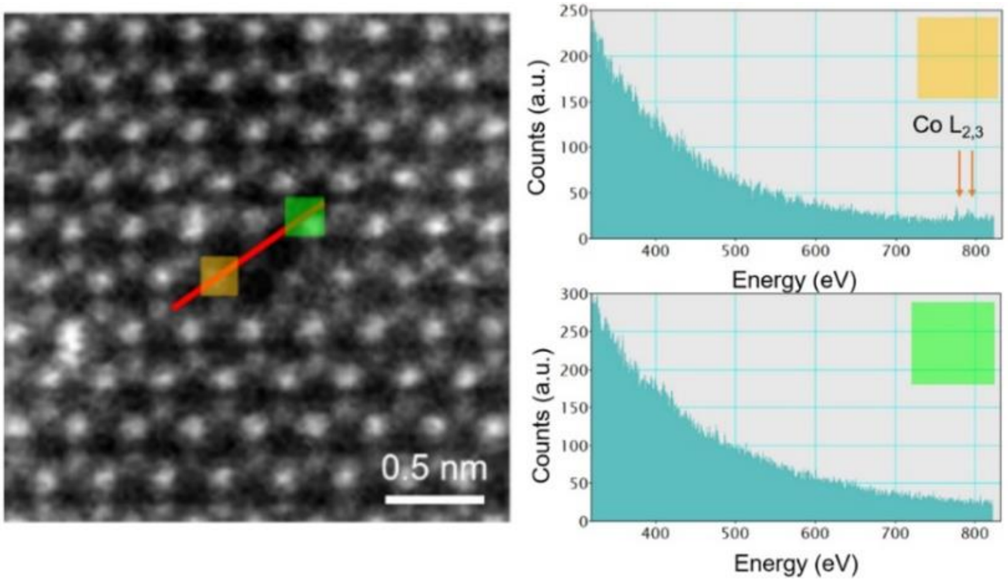

Supplementary Figure 18. EELS spectrum in a line scan collection in Co-doped MoS_2. The spectrum was collected along a line across the dopant. A sharp peak at 770 eV appeared if we integrated the EELS signal only on the dopant position, while a clean background was found when integrated far away from the dopant (the main peaks of Mo and S are at 55 eV and 167 eV, respectively, outside the energy region that collected).

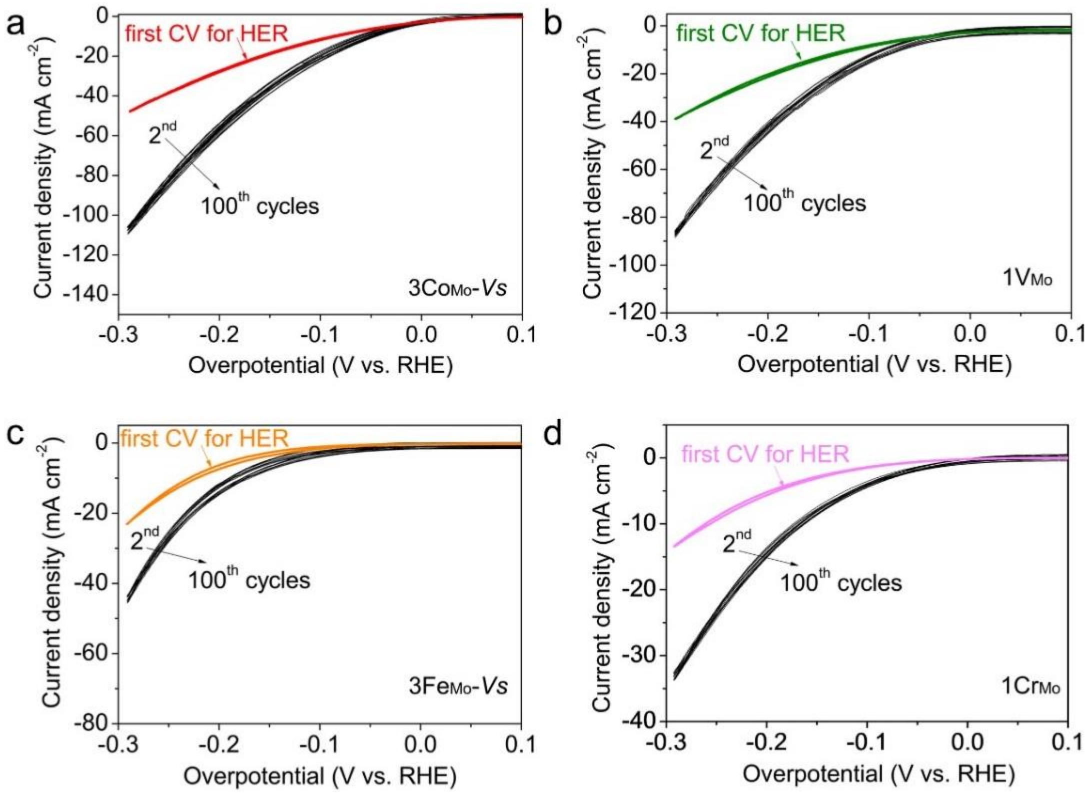

Supplementary Figure 19. The CVs during activation process (black curves) of every 10 cycles from the 2nd to 100th cycles. The first cycles of CV for the HER with rotation at 1500 rpm of a) $3Co_{Mo}$-*Vs*, b) $1V_{Mo}$, c) $3Fe_{Mo}$-*Vs*, and d) $1Cr_{Mo}$. The scan rate for activation was 100 mV s^{-1} and 2 mV s^{-1} for the first HER CVs.

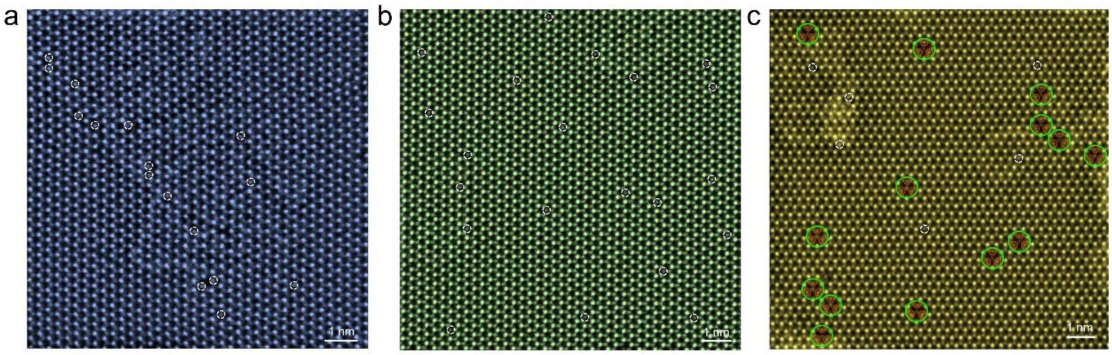

Supplementary Figure 20. Low-magnified STEM images of three types of doped samples. (a) $1Cr_{Mo}$, 10 nm×10 nm; (b) $1V_{Mo}$, 12 nm×12 nm; and (c) $3Fe_{Mo}$-*Vs*, 12 nm×12 nm. The $1TM_{Mo}$ sites are highlighted in white circles and $3TM_{Mo}$-*Vs* configurations are labeled with green circles.

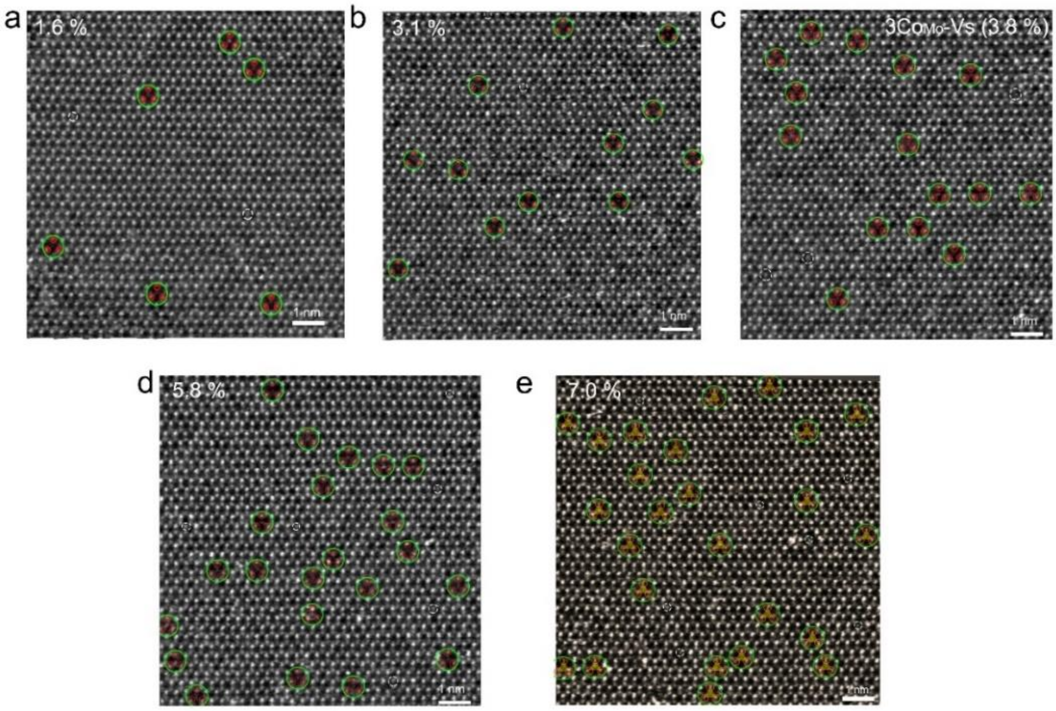

Supplementary Figure 21. Atomic STEM images of the $3Co_{Mo}$-*Vs* configured MoS_2 samples. Samples with Co concentrations of (a) 1.6 %, (b) 3.1 %, (c) 3.8 at%, (d) 5.8 at% and (e) 7.0 at%. The $3Co_{Mo}$-*Vs* configurations are highlighted in green circles while $1Co_{Mo}$ is marked by white circles. All samples are imaged at 10 nm×10 nm.

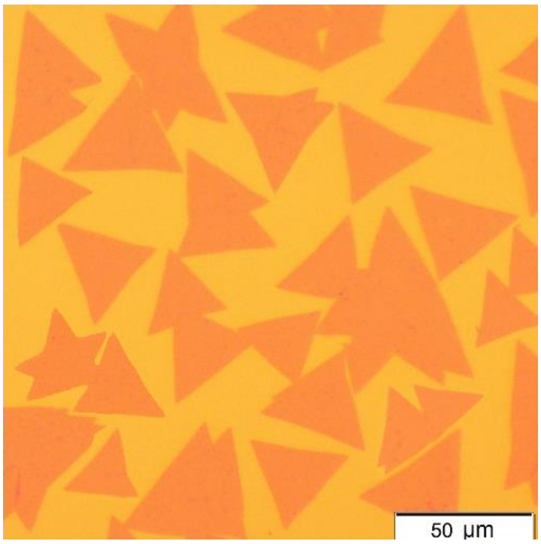

Supplementary Figure 22. Optical image of pristine MoS_2 monolayer supported on Si/SiO_2 substrate.

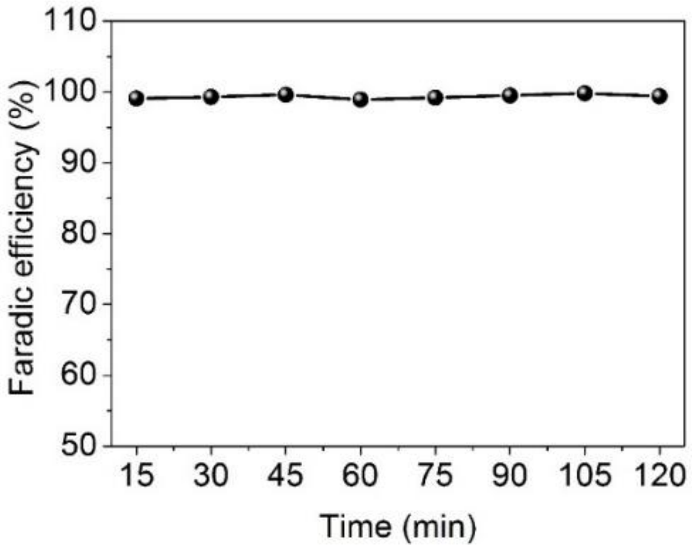

Supplementary Figure 23. Faradic efficiency obtained by measuring the amount of H_2 generated during the hydrogen evolution reactions catalyzed by the sample with $3Co_{Mo}$-*Vs* configurations at a constant potential of -0.1 V vs. RHE.

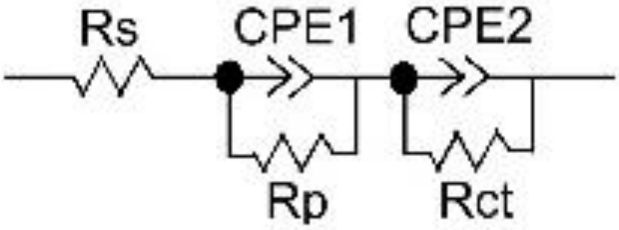

Supplementary Figure 24. The circuit model for fitting electrochemical impedance spectroscopy.

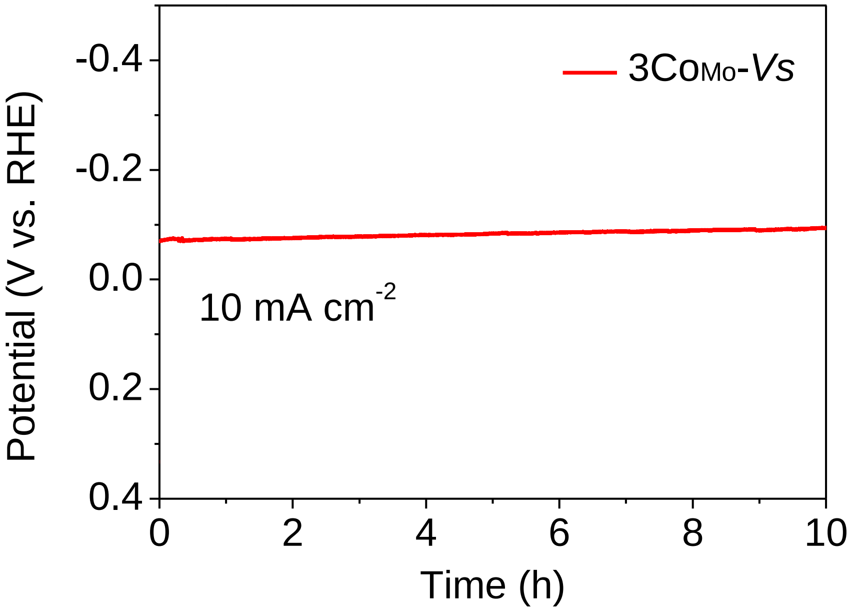

Supplementary Figure 25. Long-term HER stability of the $3Co_{Mo}$-*Vs* sample at a constant current of 10 mA/cm^2.

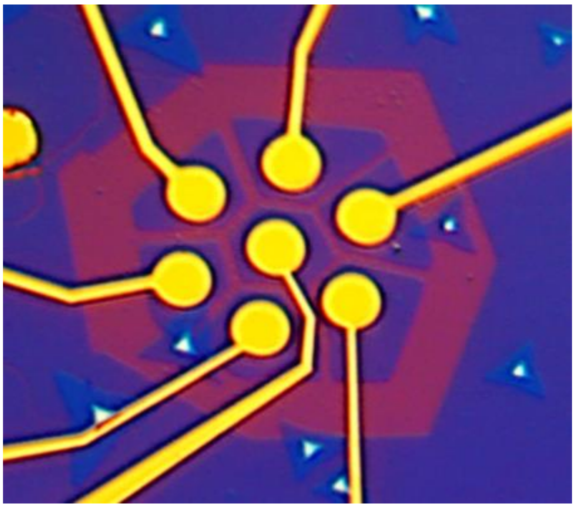

Supplementary Figure 26. The optical image to represent the multi-pads device of microcell for the sample with a specific configuration ($3Co_{Mo}$-V_S) concentration. Multi-pads used here aims to guarantee the stable testing results in the different areas of one sample.

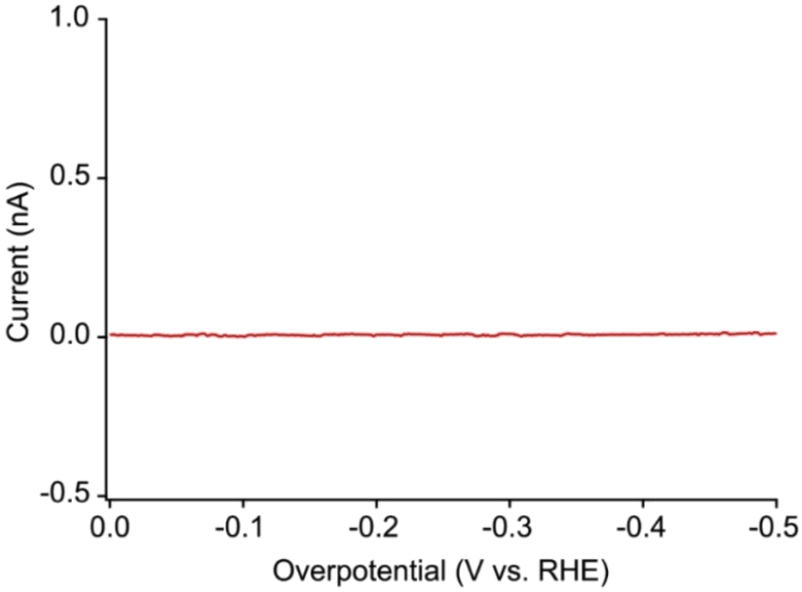

Supplementary Figure 27. Controlled I-V scan.

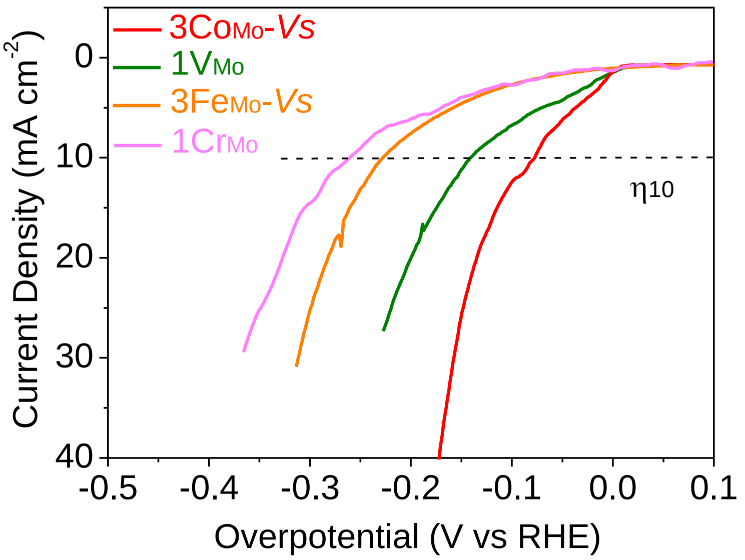

Supplementary Figure 28. Microcell HER measurements of the $3Co_{Mo}$-*Vs*, $3Fe_{Mo}$-*Vs*, $1V_{Mo}$, and $1Cr_{Mo}$ samples with ~1.2 % configuration concentrations.

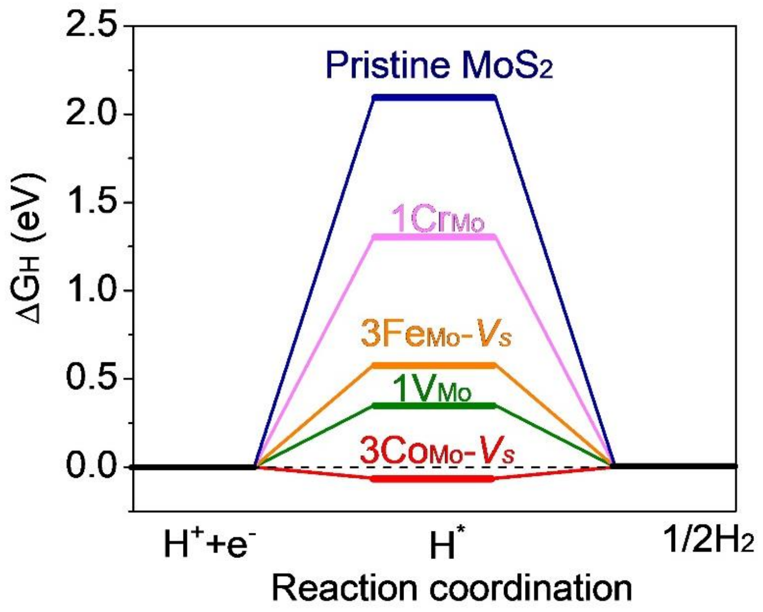

Supplementary Figure 29. The supercell convergence test. The free energy diagram for (5 × 5 × 1) supercell with $1V_{Mo}$, $1Cr_{Mo}$, $3Fe_{Mo}$-*Vs* and $3Co_{Mo}$-*Vs*. The free energy difference compared with (4 × 4 × 1) supercell is small.

Supplementary Tables

Supplementary Table 1. Hydrogen adsorption free energies (ΔG_H) for specific local configurations in Figure S2.

Configuration types	Co	Fe	V	Cr
$1TM_{Mo}$	-0.300	0.258	0.270	1.260
$1TM_{Mo}$-*Vs*	0.906	0.992	0.234	0.730
$2TM_{Mo}$	-1.043	0.290	0.116	1.267
$2TM_{Mo}$-*Vs*	0.699	0.544	0.316	0.464
$3TM_{Mo}$	-0.154	0.694	0.118	1.148
$3TM_{Mo}$-*Vs*	-0.085	0.612	0.689	0.149

Supplementary Table 2. Comparison of HER activity of MoS_2 in this work ($3Co_{Mo}$-*Vs*) with mono/few-layered TMDs and some bulk TMDs electrocatalysts measured in 0.5 M H_2SO_4.

Sample ID	Overpotential at 10 mA cm^{-2} (η_{10}, mV vs. RHE)	ΔG_H (eV)	TOFs (s^{-1})	Ref.
**Monolayered or few-layered TMDs**				
**$3Co_{Mo}$-*Vs***	**-75**	**0.048**	**3-50**	**This work**
Strained Vs-MoS_2	-170	0.08	0.05-0.16	[1]
20 nm 2H-TaS_2	~-200	-0.04	--	[2]
V_{Re}-ReS_2	-147	0.016	1-10	[3]
1T-MoS_2	<-200	~-0.15	0.2-0.5	[4]
Vs-MoS_2	<-300	~0.025	--	[5]
MoS_2 on Au	<-200	--	--	[6]
2H MoS_2 Mo edge	-201	~0.125	3.8	[7]
Vs-MoS_2 nanocrystals	~-150	--	--	[8]
2H MoS_2 basal plane	-425	--	1.9×10^{-4}	[7]
1T'-MoS_2 Mo edge	-77	--	3.8	[7]
MoS_2	~-600	--	--	[9]
**Bulk TMDs and other catalysts**				
Pd-MoS_2	-89	-0.02	--	[10]
H-TaS_2	-60	~0.01	--	[11]
H-NbS_2	-50	~-0.01	--	[11]
TaS_2 200 nm in thickness	-150	0.02	--	[2]
rGO/$W_xMo_{1-x}S_2$	-233	--	--	[12]
1T-$MoSe_2$ nanosheets	-152	--	--	[13]
Co-doped MoS_2	-156	--	--	[14]
VS_2 crystal	-68	--	--	[15]
MoS_2 on N-carbon nanoboxes	-165	--	--	[16]
ReS_2 on Au foil	~-190	--	--	[17]
$NiCo_2P_x$ nanowires	-104	--	0.021	[18]
Mo_2C@N-carbon	-74	--	--	[19]

Supplementary Table 3. The summary of domains on η_{10}, Tafel slopes, Co and corresponding $3Co_{Mo}$-*Vs*, $1Co_{Mo}$ concentrations.

	η_{10} (mV)	Tafel slopes (mV dec^{-1})	Co (%)	$3Co_{Mo}$-*Vs* (%)	$1Co_{Mo}$ (%)
1	279	147	1.6	0.5	0.2
2	184	132	3.1	1.0	0.2
3	69	52	3.8	1.2	0.2
4	220	106	5.8	1.8	0.4
5	248	117	7.0	2.1	0.6

Supplementary Notes

Supplementary Note 1

Setting one S as the center, TM atoms replace any of three connected Mo atoms shows significant change toward electronic structures of central S. As a result, the influential and possible TM atom number should be one to three as shown in Supplementary Figure 1. Based on this, the sulfur vacancy is inserted into the basal plane to form $1TM_{Mo}$-*Vs*, $2TM_{Mo}$-*Vs*, and $3TM_{Mo}$-*Vs* configurations. For V and Cr, the local structures compared to original MoS_2 lattice are less distorted; while the TM-S bonds are more elongated for Fe- and Co-containing MoS_2.

Supplementary Note 2

The illustration of the nearest atoms and the next-neighbor atoms of adsorption S1 site is shown in the Supplementary Figure 6. Based on the distance from the S1 atom, the introduced transition metal atoms and the nearest Mo atoms are defined as the nearest atoms, which are indicated by red dash circles. The next-neighbor atoms are indicated by green dash circles including six S atoms and three Mo atoms.

Supplementary Note 3

The high-resolution Co 2p and Fe 2p spectra with fitting are shown to verify the contributions of $3TM_{Mo}$-*Vs* and $1TM_{Mo}$ (Supplementary Figure 17). The absence of central sulfur atom in $3TM_{Mo}$-*Vs* configurations creates more uncoordinated (TM) atoms compared to $1TM_{Mo}$. As a result, the TM 2p spectra of $1TM_{Mo}$ should shift to higher BE position[20] compared to that of $3TM_{Mo}$-*Vs*, which is consistent with our fitted curves. In addition, the area ratio of $1TM_{Mo}$ to $3TM_{Mo}$-*Vs* is 0.1, which implies the $3TM_{Mo}$-*Vs* configuration is the dominant populations.

Supplementary Note 4

The reference EELS spectra in Supplementary Figure 18 have no sharp feature of

detected element, confirming the S/N ratio is far beyond the detection limit and the observed sharp peak in Figure 2 is not an artifact during the collection at the dopant site. Other configured samples exhibit the similar behavior.

Supplementary Note 5

Note that the current densities (*i*) of first CVs of HER with a scan rate (ν) of 2 mV s^{-1} is nearly one half or one third those of in activation CVs scanned at 100 mV s^{-1} (Supplementary Figure 19). Theoretically, the *i* of CV is proportional to the square root of ν, meaning that the values of *i* at 2 mV s^{-1} should be one seventh that at 100 mV s^{-1}. This discrepancy can be explained by the fact that we applied rotation during the measurement of first cycle CV but not for the activation, and rotation can sharply increase the current density values of *i*.

Supplementary Note 6

In order to further verify the uniformity and concentrations of dopants, the low-magnified STEM images of samples $3Fe_{Mo}$-*Vs*, $1V_{Mo}$, and $1Cr_{Mo}$ are presented (Supplementary Figure 20). It is clearly seen that most of the TM dopants are uniformly distributed throughout the lattice. For the $3Fe_{Mo}$-*Vs*, the overall Fe concentration is estimated as 3.3 %. The $3Fe_{Mo}$-*Vs* configuration accounts for over 90 %. In the sample $1V_{Mo}$, the overall concentration of V atoms (highlighted in white circles) is estimated as 1.4 %, and all corresponds to single V_{Mo} configuration. The sample $1Cr_{Mo}$ has a Cr concentration of 1.2 %.

Supplementary Note 7

Large area STEM images of the Co-doped MoS_2 monolayer are imaged at 10 nm×10 nm. The overall Co concentration is estimated. Among them, the $3Co_{Mo}$-*Vs* configuration accounts for over 90 % in all Co-doped samples. As for the sample with the best HER activity (Supplementary Figure 21c), the Co concentration is 3.8 at% with 97% of $3Co_{Mo}$-*Vs* configurations.

Supplementary Note 8

The HER is generally accepted to proceed via the steps (Equation 1-3) noted below, where in the initial proton discharge to form adsorbed H (Equation 1) is followed by either the recombination of the adsorbed H to form H_2 (Equation 2) or electrochemical desorption of the adsorbed intermediate to form H_2 (Equation 3).

$$M + H^+ + e^- \Leftrightarrow MH \text{ (Tafel mechanism)} \quad (1)$$

$$MH + MH \Leftrightarrow H_2 + 2M \text{ (Heyrovsy mechanism)} \quad (2)$$

or

$$MH + H^+ + e^- \Leftrightarrow H_2 + M \text{ (Volmer mechanism)} \quad (3)$$

Supplementary Note 9

The turnover frequency is calculated using the current density j and the active site density N according to Equation 4,

$$TOF = \frac{\text{Total number of } H_2 \text{ atoms per second}}{\text{Total number of active sites per unit area}} = \frac{j/(2 \times q)}{N} \quad (4)$$

Where q = 1.6×10^{-19} C is the elementary charge, and 2 accounts for 2 H atoms per H_2 molecule. To calculate the turnover frequency per surface S atom (TOFs), the S atom density (N_S) is estimated to be 2×10^{15} cm^{-2} from the MoS_2 lattice constant ~3.2 Å.

Supplementary References

1. Li H., *et al.* Activating and Optimizing MoS_2 basal planes for hydrogen evolution through the formation of strained Sulphur vacancies. *Nat Mater* **15**, 48-53 (2016).

2. Shi J., *et al.* Two-dimensional metallic tantalum disulfide as a hydrogen evolution catalyst. *Nat Commun* **8**, 958 (2017).

3. Zhou Y., *et al.* Auto-optimizing hydrogen evolution catalytic activity of ReS_2 through intrinsic charge engineering. *ACS Nano*, **12**, 4486-4493 (2018).

4. Voiry D., *et al.* The role of electronic coupling between substrate and 2D MoS_2 nanosheets in electrocatalytic production of hydrogen. *Nat Mater* **15**, 1003-1009 (2016).

5. Tsai C., *et al.* Electrochemical generation of sulfur vacancies in the basal plane of MoS2 for hydrogen evolution. *Nat Commun* **8**, 15113 (2017).

6. Shi J., *et al.* Controllable growth and transfer of monolayer MoS_2 on Au foils and its potential application in hydrogen evolution reaction. *ACS Nano* **8**, 10196-10204 (2014).

7. Zhang J., *et al.* Unveiling active sites for the hydrogen evolution reaction on monolayer MoS_2. *Adv Mater* **29**, 1701955 (2017).

8. Lin L., *et al.* Sulfur-depleted monolayered molybdenum disulfide nanocrystals for superelectrochemical hydrogen evolution reaction. *ACS Nano* **10**, 8929-8937 (2016).

9. Ye G., *et al.* Defects engineered monolayer MoS_2 for improved hydrogen evolution reaction, *Nano Lett.* **16**, 1097-1103 (2016).

10. Luo Z., *et al.* Chemically activating MoS_2 via spontaneous atomic palladium interfacial doping towards efficient hydrogen evolution. *Nat Commun* **9**, 2120 (2018).

11. Liu Y., *et al.* Self-optimizing, highly surface-active layered metal dichalcogenide catalysts for hydrogen evolution. *Nat Energy* **2**, 17127 (2017).

12. Lei Y., *et al.* Low-temperature synthesis of heterostructures of transition metal dichalcogenide alloys ($W_xMo_{1-2}S_2$) and graphene with superior catalytic performance for hydrogen evolution. *ACS Nano* **11**, 5103-5112 (2017).

13. Yin Y. *et al.* Synergistic phase and disorder engineering in 1T-$MoSe_2$ nanosheets for enhanced hydrogen-evolution reaction. *Adv Mater* **29**, 1700311 (2017).

14. Deng J. *et al.* Multiscale structural and electronic control of molybdenum disulfide foam for highly efficient hydrogen production. *Nat Commun* **8**, 14430 (2017).

15. Yuan J. *et al.* Facile synthesis of single crystal vanadium disulfide nanosheets by chemical vapor deposition for efficient hydrogen evolution reaction. *Adv Mater* **27**, 5605-5609 (2015).

16. Yu X. *et al.* Ultrathin MoS_2 nanosheets supported on N-doped carbon nanoboxes with enhanced lithium storage and electrocatalytic properties. *Angew Chem Int Ed* **54**, 7395-7398 (2015).

17. Gao J. *et al.* Vertically oriented arrays of ReS_2 nanosheets for electrochemical energy storage and electrocatalysis. *Nano Lett* **16**, 3780- 3787 (2016).

18. Zhang R. *et al.* Ternary $NiCo_2P_x$ nanowires as pH-universal electrocatalysts for highly efficient hydrogen evolution reaction. *Adv Mater* **29**, 1605502 (2017).

19. Ma R. *et al.* Ultrafine molybdenum carbide nanoparticles composited with carbon as a highly active hydrogen-evolution electrocatalyst. *Angew Chem Int Ed* **54**, 14723-14727 (2015).

20. Li L., *et al.* Role of sulfur vacancies and undercoordinated Mo regions in MoS_2 nanosheets toward the evolution of hydrogen. *ACS Nano* **13**, 6824-6834 (2019).
